# Plant Extract Based on Deep Eutectic Solvent-Mediated Biosynthesis of Silver Nanoparticles: Cytotoxicity and Antibacterial Effects

**DOI:** 10.1155/2023/9672432

**Published:** 2023-01-14

**Authors:** Victoria I. Vorobyova

**Affiliations:** Department of Physical Chemistry, National Technical University of Ukraine “Igor Sikorsky Kyiv Polytechnic Institute”, Kyiv 03056, Ukraine

## Abstract

Deep eutectic solvent DES-based grape pomace extracts (GPE) were used to synthesize silver nanoparticles (AgNPs) for the first time. This paper presents a synthesis of AgNPs by a novel method with GPE obtained by an eco-friendly “green” solvent, namely, betaine-lactic acid and proline-lactic acid DESs. Compared with the water-based GPE, the DES-based grape pomace extracts contain lower reducing powers but additionally act as capping agent, which is the more important property for the creation of necessary particle nanosize and dispersion with colloidal stability. DESs were synthesized using a heating method, and Fourier transform infrared spectroscopy (FTIR) was carried out to confirm the formation of the DES. The phytochemical profile of GPE exhibits a high amount of hydroxycinnamic acids (23%), followed by anthocyanins (19%). The silver nanoparticles with a round shape were noticed on the scanning electron microscopy micrographs with the particle size in the range of 10–20 nm. The disc diffusion technique (DDT) showed that the AgNPs exhibited significant antibacterial activity against Gram-negative bacteria *Escherichia coli* (*E*. *coli*) UKM В-906 and Gram-positivespore-forming*Bacillus subtilis* (*B*. *subtilis*) UCМ В-506T. Mitotic index (MI) and chromosomal aberrations (CAs) were assessed by *A*. *cepa* assay. The synthesized silver nanoparticles do not induce cytogenotoxic and genotoxic changes in *Allium cepa* L. with nanoparticles at concentrations up to 10%.

## 1. Introduction

The biosynthesis of nanoparticles has been advanced and has been an attractive field of scientific research in the last decades. Therefore, the use of green chemistry knowledge and a green approach for the synthesis of nanoparticles is steadily increasing to achieve an eco-friendly procedure. Plant extracts, bacteria, and algae have been used for the biosynthesis of metal and metal oxide nanoparticles. Among them, using the plant is a rapid, cost-effective, facile, and simple process to synthesize nanoparticles at a large level [1–7]. Moreover, green nanomaterials possess a significant application in the pharmaceutical market, such as pharmaceutical raw materials and drug delivery [8]. Silver nanodendrites (AgNPs) have multipurpose characteristics because of their large surface area to volume ratio. Various synthesis methods are reported, such as chemical vapour deposition (CVD) [9], hydrothermal [10], liquid phase reduction [11], solvothermal wafer scale manufacturing by photolithography [12], electrochemical methods [13], electrodeposition [14], metallic electromigration, galvanic displacement method [15], replacement reaction ultraviolet photoreduction technique [16], and bio-mediated synthesis [17–19]. The use of eco-friendly solvents, namely, deep eutectic solvents for the extraction of “green” reductant compounds for the synthesis of inorganic NPs are gaining popularity [20–26]. However, these methods are based on the use of toxic and aggressive organic solvents or reaction media. Even for the “green”/phytochemical synthesis of metals and metal oxide nanomaterials, “traditional” methanol, acetone, toluene, and ether plant extracts will continue to be used, which significantly limits the multifunctionality of their properties and the field of their use [27, 28]. Therefore, according to the authors, today it is not enough to modernize the above-mentioned synthesis technologies. It is necessary to use an alternative, actually biodegradable, and effective “green” solvents/reaction media/matrices with their further study and improvement, in order to establish the possibility of obtaining biocompatible/bioresistant nano-sized materials for a wide range of purposes with a simultaneous study of the patterns of influence of the method and conditions of synthesis on the properties and surface morphology of the obtained materials [28]. Some progress has been achieved in the use of environmentally safe emulsion systems for the synthesis of nanomaterials (oil/water) [29–31]. However, the technology is complicated by the specific colloidal-chemical and a molecular-kinetic property of such systems, and further development in this direction is only possible when transitioning to new types of soluble systems. For the reasons stated above, the so-calledlow-temperature eutectic solvents are attractive, generally recognized as safe in the world (generally recognized as safe), fully comply with the principles of green chemistry, and are considered 4th generation solvents for the chemical technology of the XXI century. Since 2008, among foreign scientists [21, 32–34], “green” nanotechnologies have acquired a rapid development, in which the above-mentioned deep eutectic solvents [32] have been used, which are considered as a new generation of cheap and environmentally safe ionic liquids with a number of practically useful properties, characterized by availability, variability of components, simplicity of synthesis, and a unique combination of high extractability, thermal and chemical stability, and polarity stability [21, 33, 34]. Novel green solvents called deep eutectic solvents (DESs) have recently attracted considerable attention from the scientific community in various fields. They can be prepared from two components, one as a hydrogen bond donor (HBD) and another one as a hydrogen bond acceptor (HBA). Researchers discovered that the obtained eutectic mixture had a significantly lower melting point than that of its original ingredients. Their unique attributes, such as simple preparation, low cost, nonflammability, and low toxicity, are the primary advantages over conventional volatile organic solvents and ionic liquids. The physicochemical properties and unique performance of DESs are finely tuned depending on their initial materials via different combinations and proportions of HBA and HBD. In particular, scientists in their work use DES in the synthesis of nanosized metal particles (AuNPs [22] and AgNPs [23]) and oxide compounds (MFe_2_O_3_ (M = Mg, Zn) [25, 29]; Mn_3_O_4_, and TiO_2_ [26]) of different component compositions and materials based on them for use as sorption-photocatalysts, sensor materials, and nanofluids. It should be noted that DESs are used exclusively as a reducing reagent/reaction medium or stabilizer [22, 23, 25, 26, 29]. At the moment, there are several works in the literature on the synthesis of silver nanoparticles using a plant extract based on DESs. Thus, four different solvents were used to extract tea leaves and further synthesize silver nanoparticles (betaine-glycerol; glycerol/betaine/glycerol- urea) [35]. There is a lack of attention and reports on aspects of the synthesis of silver nanoparticles by the extracts obtained using deep eutectic solvents (DES). Thus, considering the lack of scientific development on truly “green” methods for the synthesis of nanodispersed and nanostructured oxide materials, the authors propose conducting research on the use of deep eutectic/natural deep eutectic solvents as extractants of reducing organic compounds for phytochemical/biomimetic synthesis. There are publications in the literature that describe the use of aqueous extracts of grape pomace for the synthesis of silver nanoparticles [36, 37]. However, the resulting systems have low colloidal stability and do not have a significant shelf life due to the ability of the extracts to quickly turn sour.

In previous work, the formulation and physicochemical properties of betaine-based and proline-based natural deep eutectic solvents were researched [38]. When using DES for the synthesis of nanoparticles, it is important that they are stable when mixed with water, since ionic liquids must retain the formed hydrogen bonds. In an earlier publication, detailed studies were carried out. The effect of water content on the redox properties of the DES was studied by cyclic voltammetry. The width of the electrochemical stability window decreases with the increase in water content. The presence of water will increase the extractability of the solvents. Previously, choline chloride-based deep eutectic solvents had already been performed for the extraction of “green” compounds from tomato pomace [39]. The water content has been shown to be a positive factor.

In this work, it is proposed to compare the physicochemical properties of silver nanoparticles obtained by synthesis using a traditional water extract of grape pomace (W-GPE) and based on a betaine/proline-based natural deep eutectic (DES-GPE) solvent. In this work, the application of betaine-based natural deep eutectic solvents as green extraction solvents was proposed for the first time for the extraction of “green” reductant compounds intended to be applied for the synthesis of silver nanoparticles.

## 2. Materials and Methods

### 2.1. DES Characterization

DES was prepared according to the procedure described in previous works [38–40]. For obtaining the liquid eutectic system at room temperature, the HBA/HBD molar ratios of these mixtures were kept at 1 : 2 in the next characterization processes (Table 1).

Lactic acid and betaine were weighed, as required to achieve the molar ratio of 1 : 2 and mixed in a 200 mL flask. DES was synthesized by heating (at 60°C (water bath)) of lactic acid and betaine. The obtained DES was used as an extractant for extracting of organic compounds from grape pomace, with the following use in the synthesis of the NP procedure without any further purification.

### 2.2. Extraction Procedure

Two types of extracts were prepared. The grape pomace powder was added into DES (betaine- or proline-lactic acid) in a solvent/solid ratio of 10/1. This mass/solvent ratio was selected after preliminary studies to favor the extraction while assuring the plant material is completely covered by the solvent used (DES or water). The mixture was placed in the ultrasonic bath with an ultrasonic input power of 40 W and a frequency of 30 kHz. The extraction parameters were as follows: temperature 65°C, duration 60 min. Then, the extract was decanted and filtered through a paper filter. In the second case, vegetable raw materials were mixed with water. The extraction parameters were as follows: temperature 65°C, duration 60 min. The resulting extracts were named as follows: W-GPE, B-GPE, and P-GPE.

### 2.3. Extract Characterization (HPLC-DAD and HPLC-MS)

An HPLC separation method was used. Conditions of HPLC analysis: chromatographic column: Kromasil 100 C18 size 0.125 m × 4.6 mm with a particle size of 5 *μ*m (Supelco, USA). Mobile phases: *A*;0.3 g/l solution of phosphoric acid (pH 2.0), and *B*–methanol. Elution program: 0 –1 min: 60% of phase *A*; 1–20 min: 60 – 45% of phase *A*; 20–21 min: 45  – 0% of phase *A*; 21–25 min: 0% of phase *A*; 25–27 min: 0  – 60% of phase *A*; 27–35 min: 60% of phase *A*. Mobile phase speed: 1.0 ml/min. Chromatograms were obtained at profiles 325, 354, and 560 nm at the range of 200–400 nm with, diode array detector (DAD), electrospray ionization (ESI), and a quadrupole analyzer. The DAD detector scanned in the range 190–800 nm with 4 nm resolution and 640 msec sampling time. The ESI experimental conditions were: interface voltage 4.5 kV, interface temperature 350°C, desolvation line (DL) temperature 250°C, nebulizing gas flow 1.5 L/min, drying gas flow 15 L/min, and heat block temperature 200°C.

Agilent 1260 infinity series HPLC equipped with a triple quadrupole mass spectrometer (MS) with an electrospray (ESI) source operating in negative and positive ionization modes were used for HPLC-MS and HPLC-MS/MS analyses, which were controlled by Mass Hunter Workstation software. The MS analysis was as follows: MS full-scan acquisition (*m*/*z* 50–1000) in negative and positive ionization modes. Voltages for the skimmer and the capillary were, respectively, 4000 and 3500 V. Other MS conditions were as follows: nebulizer gas (*N*2), 50 psi; drying gas (*N*2), 10 L/min; dry temperature, 350°C.

Their separation was performed using a Kinetex PFP analytical column (100 mm × 2.1 mm i.d., particle size 2.6 *μ*m) from Phenomenex (Torrance, CA, USA). The mobile phase was a mixture of water (*A*) and methanol (*B*) both with formic acid 0.1% at a flow rate of 0.2 mL·min^−1^ in a gradient elution mode. The composition of the mobile phase varied as follows: 0–2 min, 20% *B*; 2–15 min, 80% *B*; 15–18 min, 80% *B*; 18–23 min, 100% *B*, 23–35 min, 20% *B*. The volume of the injection was 2 *μ*L. The temperature of the column was 30°C, while the temperature of the drying gas in the ionization source was 350°C. The nebulizer pressure was 25 psi, the gas flow was 10 L·min^−1^, and the capillary voltage was 4000 V.

The identity of compounds was ascertained using data from DAD and MS analysis, by comparison and combination of their retention times, and by UV-Vis and mass spectra and confirmed with authentic standards when available. Chromatograms were acquired at 280, 325, 354, and 560 nm.

Quantification was performed by HPLC-DAD according to an external standard method. Furthermore, the calibration curves, limits of detection (LOD), and quantification (LOQ) of the three target compounds are shown in Table 2. LOD: limit of detection; LOQ: limit of quantification. For each curve, the equation is *y* = *ax* + *b*, where *y* is the peak area, *x* is the concentration of the analyte (*μ*g/mL), *a* is the slope, *b* is the intercept, and *R*^2^ the coefficient of correlation.

### 2.4. Antioxidant Capacity Determination

The scavenging of 2, 2-diphenyl-1-picrylhydrazulin (DPPH) radicals and 2, 2′-Azino-bis-(3-ethylbenzothia-zoline-6-sulf-onic acid) (ABTS) were performed. The antioxidant activity was measured using the ferric-reducing antioxidant power (FRAP) method as described by Sánchez-Moreno, Larrauri, and Saura-Calixto. Absorbance was measured at 700 nm and the results. All determinations were performed in triplicate.

### 2.5. Total Polyphenol and Flavonoid Contents in the Extracts

A total polyphenol and flavonoid content was determined using classic methods. The content of the amount of flavonoids were determined by the spectrophotometric method in terms of quercetin. The content of the sum of phenolic compounds was determined by the spectrophotometric method uses the Folin–Ciocalteu reagent method in terms of gallic acid. To do this, 0.2 cm^3^ of the obtained extract was transferred to a 25 cm^3^ volumetric flask, 17.5 cm^3^ of buffer solution (pH = 12.9), 1 cm^3^ of Folin–Ciocalteu reagent was added and brought to the mark with purified water. The contents of the flask were mixed and left for 30 min. The optical density of the obtained solution was determined on a spectrophotometer at a wavelength of 760 nm in a cuvette with a layer thickness of 10 mm. A mixture consisting of 1 cm^3^ of Folin–Ciocalteu reagent, 17.5 cm^3^ of glycol buffer solution with pH = 12.9, and 6.5 cm^3^ of purified water was used as a comparison solution. In parallel, the optical density of a solution of a standard sample of gallic acid prepared similarly to the investigated solution was determined.

### 2.6. Synthesis and Identification of Silver Nanoparticles

In this study, grape pomace extract based on DES and water was used to synthesize AgNPs. The equivalent volumes of the extract solution and 0.01 M AgNO_3_ were heated separately in the glass beakers in the water bath to a temperature of 40°C. Then, the extract solution was added to the AgNO_3_ and left on a water bath for 15 min. The biosynthesized AgNPs were identified by a fully spectroscopic study. A Hitachi U-2900 spectrophotometer was used for UV-Vis measurement. The AgNPs were characterized by SEM. The surface morphology of the prepared AgNPs was evaluated using scanning electron microscopy. A drop of colloidal system was applied on the carboncoated copper plate, and an image was taken at an acceleration voltage of 10 kV (CamScan with an X-ray attachment for microanalysis INCA-200 energy).

### 2.7. *Allium cepa* Root Growth Inhibition Test

To study the antimitotic and cytotoxicity, the Allium test was chosen, which is recommended by experts of the World Health Organization as a standard in cytogenetic monitoring [41]. The object of research in this test is the meristem of the roots of *Allium cepa* (onion) bulbs—*Allium cepa* of the Stut-Garten-Riesen variety. This method is simple, economical, and short-term, recommended in ecological-genetic studies by a group of experts, and sensitive enough to determine “mutagen” or “nonmutagen.” The onion bulbs of approximately equal size and with no damage of pests and bacterial or fungi diseases were growing in plastic glasses filled with test solutions for 4 days (96 hours). Seven experimental variants were carried out, corresponding to concentrations of the test solutions:

- Control (distilled water)
- Water solution of deep eutectic solvents in concentrations of 1%, 5%, and 10%
- Solution of AgNPs in concentrations of 1%, 5%, and 10%

All patterns of solutions were executed in three repetitions. At the end of the growing period, the onion bulbs were removed from the test solutions. The fixation of root meristematic cells was carried out, followed by visual microscopic analysis, and mitotic index calculation.

#### 2.7.1. Fixation and Coloring of Onion Roots

The roots normally grew to a length of about 1 cm, then they were cut and fixed overnight in a fridge at 4°C in Clark's fixative solution (two parts of 96% ethyl alcohol and one part of glacial acetic acid). After that the roots were hold for 5– 6 minutes in a 1 M hydrochloric acid solution. Next, was the dyeing procedure: roots were immersed in an acetocarmine solution for 60 minutes and washed in 45% acetic acid for 15 minutes. The acetocarmine solution was prepared by the dissolution of 1 g of carmine in 100 ml of 45% acetic acid. Before the test, the filtered solution was stored in the dark.

The roots were removed from the solution and made prepared for microscopy according to the method of flattening the root tip to obtain cells with good visualization. For this purposes, a small tip of the root (only the meristem tissue) was cut off, placed on a glass slide, covered with a cover slip, and lightly squashed by pressing.

#### 2.7.2. Microscopic Observation and Calculation

About 200 cells of each variant were analyzed by an optical microscope with ×1000 magnification. The vital cellular activity was monitored by the estimation of vital cells quantity and calculation of the mitotic index. Cells viability was determined by the state of the membranes, cytoplasm, and nucleus and the absence of cellular abnormalities. The mitotic index (MI, %) was calculated as the total number of cells divided by the total number of cells including cells in interphase the following equation:

(1) $$\begin{array}{c} mitotic\;index\left(MI\right)=\frac{\left(P+M+A+T\right)}{\left(total\;number\;of\;cells\right)}\;∙\;100\%\text{.} \end{array}$$

The stages of cell division are *P*-prophase, *M*-metaphase, and *A*-anaphase*N*-telophase.

The assessment of the MI difference validity between samples and control was carried out by the formal statistical method using the Student's *t*-test in a Microsoft Excel environment.

### 2.8. Antibacterial Activity

Test strains, such as Gram-negative bacteria *Escherichia coli* (*E*. *coli*) UKM В-906 and Gram-positivespore-forming*Bacillus subtilis* (*B*. *subtilis*) UCМ В-506T were received from the Danylo Zabolotny Institute of Microbiology and Virology of the National Academy of Science of Ukraine. The antibacterial performance of the biosynthesized AgNPs was studied against Gram-positive and Gram-negative bacteria based on the disc diffusion technique (DDT).

Before testing inoculums were cultivated in a liquid medium of meat peptone broth during for 6 hours at 37°C. Inoculum suspension was diluted up to an optical density of 0.5 on the McFarland standard scale that corresponds to 10^8^colony-forming units (CFU) per cm^3^.

The potential of nanoparticles regarding the antimicrobial activity was determined using the disk diffusion method (as described in CLSI Clinical and Laboratory Standards Institute: M02-A11 standard (Performance Standards for Antimicrobial Disk Susceptibility Tests), by impregnating AgNP-containing solution on each 6 mm diameter disk made of glass microfiber filters from Whatman TM.

The inoculum was densely touched by a swab on sterile agar in Petri dishes, and after that the pieces of catgut threads were placed on the surface. Petri dishes were incubated at 37°C for 24 and 48 hours. Evaluation of antibacterial activity was provided by measuring the size of the inhibition zones around the threads.

Antibacterial action was estimated for such samples:

- Control: medical catgut in sterile solution
- *N* 1 (*e*): medical catgut with Ag after electrophoresis
- *N* 2 (*x*): medical catgut macerated in Ag-NPs solution (chemical method)

In this study solution distilled water and an antibiotic (aztreonam) were used as a control. Disks containing aztreonam 5 g was used as control. The whole set of measurements was performed in triplicate and the averaged values were reported.

### 2.9. Statistical Analysis

Averaged result of three replicates for each sample was used for the subsequent statistical analysis. The linear regression model was proposed by using the software MATLAB.

## 3. Results and Discussion

### 3.1. Characterization of the Grape Pomace Extract Obtained by Deep Eutectic Solvents

**Figure 1:**
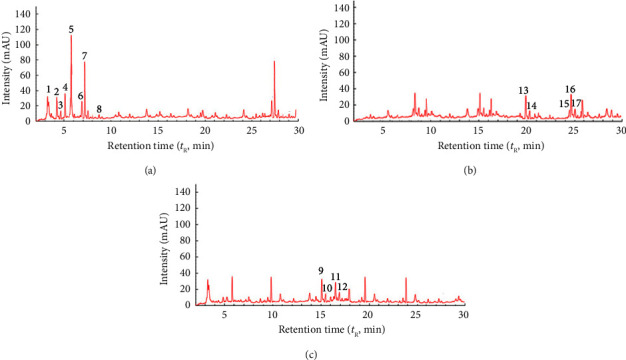
HPLC profiles at 325 nm (a), 354 nm (b), and 560 nm (c) of the grape pomace extract obtained using water.

**Figure 2:**
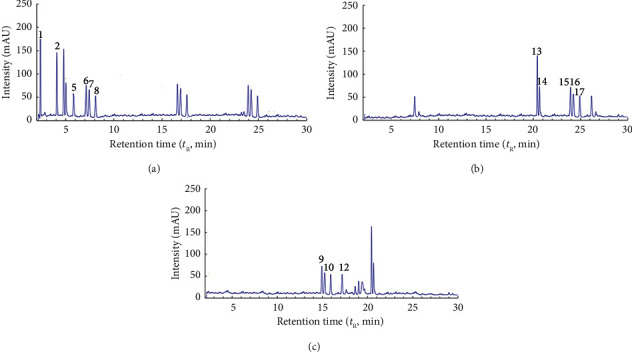
HPLC profiles at 325 nm (a), 354 nm (b), and 560 nm (c) of the grape pomace extract obtained using the proline-based eutectic solvent (DES-1).

**Figure 3:**
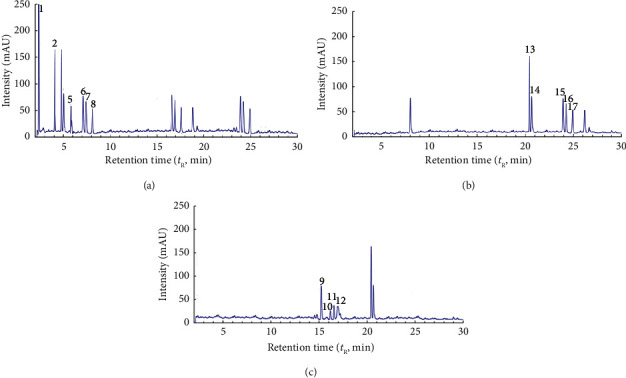
HPLC profiles at 325 nm (a), 354 nm (b), and 560 nm (c) of the grape pomace extract obtained using the betaine-based eutectic solvent (DES-2).

Figure 1 shows chromatograms for GPE obtained by water. Figures 2 and 3 show chromatograms for GPE obtained by DES (Tables 3 and 4). A comparative analysis of the results of the chromatographic analysis showed no pronounced changes in the qualitative composition of the samples. According to the present study, the main components of water GPE samples bitter are hydroxycinnamic acids and anthocyanin. It was established that the higher the content of flavonoids and anthocyanin is observed in DES solvents. According to the results of the study of the influence of the type of solvent on the content of phenolic compounds, the maximum content of anthocyanins was observed in the extract obtained by DES-1. The sample extracts were analyzed by the HPLC method with the use of the mass detection method, and it was found that the phenolic acids are represented by compounds: gallic acid, protocatechuic acid, p-hydroxybenzoic acid, coutaric acid, caffeic acid, epicatechin, syringic acid, and p-coumaric acid. When using the solvent water, the extraction of hydroxycinnamic acids and flavonoids is observed; therefore, it was used as a reference solvent to determine the effectiveness of the use of new solvents. Predictably, when water and ethanol are used, a reduced amount of flavonoids can be expected, since a solvent with a neutral pH ensures the extraction of more polar compounds. The obtained results confirm that when using a water alcohol solution, hydroxycitric acids are identified to a greater extent, while flavanols and flavonoids are less abundant. The obtained results are in good agreement with the literature data regarding the phenolic composition of grape pomace [36].

Grape pomace extract mainly consists of phenolic compounds, predominantly gallic acid (10.8%) and caffeic acid (8.7%). These compounds are known today for their reducing ability. The dominant anthocyanins were identified as petunidin 3-O-glucoside and delphinidin 3-O-glucoside. Thus, the analysis of the composition of the grape pomace extracts indicates that it contain organic substances and therefore are a potential raw material for use as a reducing and capping agent and are especially promising for producing nanoscale material.

The results of the determination of polyphenols by the Folin–Ciocalteu reagent method showed a high content of polyphenols in the selected extracts. The determined content of polyphenolic compounds is close between samples for three solvents (Table 5). The highest content of polyphenols was found in the extract obtained by the DES-1, and the value was higher than 90 mg gallic acid equivalent/g of the dry fraction of the extract. The extract obtained with a water-ethanol solution was distinguished by a significantly lower content of polyphenols of approximately 45.10 ± 1.75 90 mg gallic acid equivalent/g of the dry fraction. These data indicate that the applied DESs as solvents are a good method one production of extracts with a high concentration of polyphenols. Significantly higher content of flavonoids was observed with the DES-1 system (51.36 ± 1.20 mg QUE/g d.w.) in comparison with the reference solvent (water) (12.15 ± 1.20 mg QUE/g d.w.). The qualitative content of polyphenols (hydroxycinnamic acids, flavonols, and anthocyanins), which was determined by the method of high-performance liquid chromatography in the extracts obtained from grape pomace, is presented in Table 5. The extracts obtained by DES-1 and DES-2 contained the greatest amount and variability of polyphenols (which was also confirmed by the Folin–Ciocalteu reagent analysis method). The main component of the DESs was gallic acid with a concentration of 792–825 *μ*g/g. The results obtained here are consistent with those of a previous study suggested that the more effective solvent for the extraction of phenolic compounds cooperated with organic solvents is a deep eutectic solvent.

**Figure 4:**
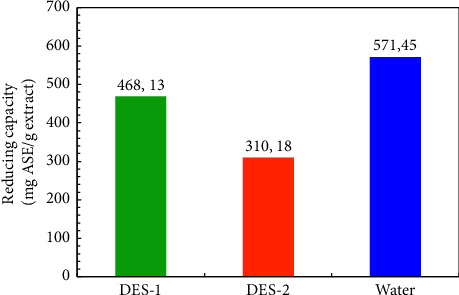
Reducing powers of the grape pomace extracts were evaluated by the phosphomolybdenum method.

**Figure 5:**
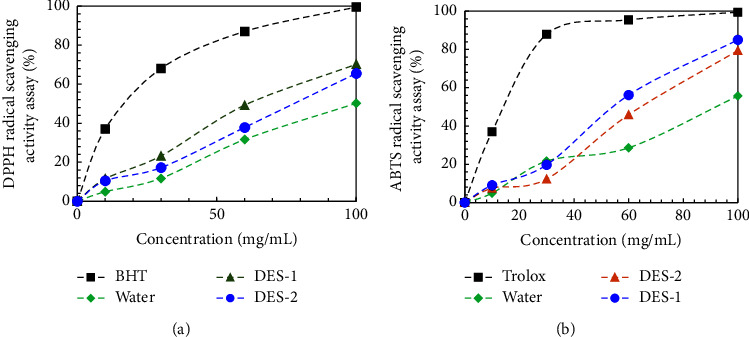
DPPH (a) and ABTS (b) radical scavenging activity of the grape pomace extracts.

**Figure 6:**
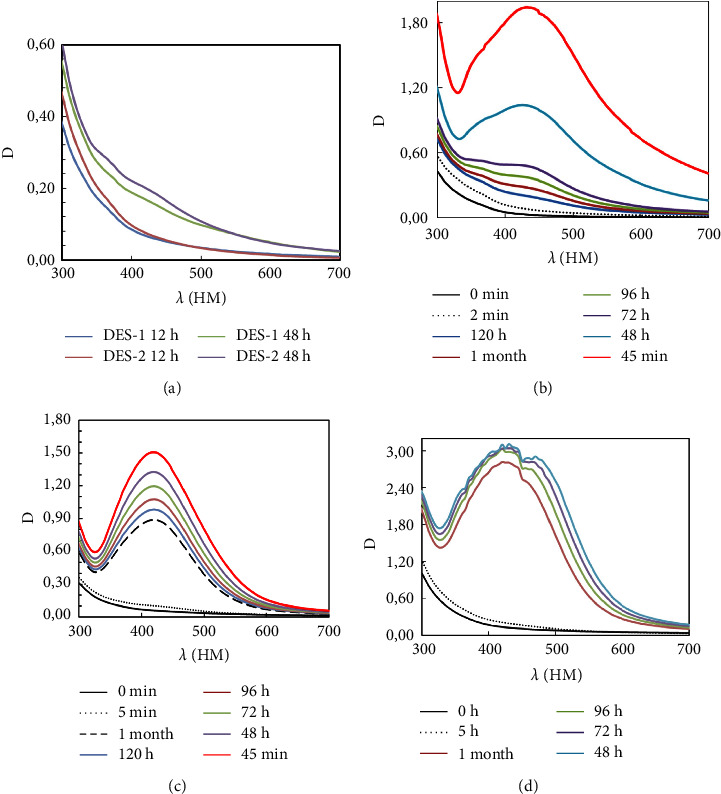
UV-visible spectra of AgNO_3_ with only DESs (a) in water and “green” synthesized silver nanoparticles with grape pomace extract obtained by P-GPE (b), B-GPE (c), and W-GPE (d).

To better understand how component composition of extract effects on the synthesis, reducing the power of the grape pomace extracts was compared. Thus, it can be concluded that the reducing ability of the studied extracts is sufficient for the reduction of silver ions (Figure 4). Since real objects are rather complex systems in terms of chemical composition, the problem of the practical use of antioxidants of plant origin is the quantitative assessment of their antioxidant activity, which is realized due to the total content and action of reducing agents of various natures. In addition, when assessing the antioxidant capacity, it is necessary to take into account not only the nature and content of reducing agents in the object under study, but and the possibility of their mutual influence. Figures 5 and 6 show the results for variation in antioxidant capacity with the type of DESs that were used for GPE extraction. Analyzing the data obtained, it can be noted that there is a correlation between the AOC of the samples of medicinal plants and the total content of all phenolic compounds in them: hydroxycinnamic acids, anthocyanins, anthoxanthins, and stilbenes (Figure 5). Water maceration was used as a reference extraction. This value is represented in Figure 5 as a horizontal line, and it can be seen that DES extractions yielded significantly higher levels of reducing power, which means the antioxidant capacity (AOC) than maceration with a solution of water.

In Figure 5, it can be seen that extraction with DESs-1 also had higher AOC values evaluated by DPPH and ABTS methods than extraction with DESs-2 and maceration with, water implying a favorable effect of that solvent on compound extraction. The best result from the approximation of experimental data was obtained using the polynomial dependence. The method evaluated for AOC did not have a significant effect on the extracts' AOC, according tour results. In the absence of an inhibitor, the ABTS molecule is oxidized by potassium persulfate, and an increase in absorption in the spectrum regions characteristic of ABTS^•+^ is recorded. In the presence of an antioxidant, there is an instant reduction of the radical cation with the formation of the original ABTS molecule. Our results for ABTS are higher than those reported by these authors, and with the advantage of having been obtained in a shorter time using intact grape pomace and a more diluted system [30, 42].

### 3.2. Characterization of AgNPs Synthesized by the DES-Based Grape Pomace Extract

The negative control, in which AgNO_3_ was mixed with only DESs in water, showed no color change or surface plasmon resonance (SPR) band (data not shown). The synthesized DES systems and their components were used for the synthesis of colloidal silver nanoparticles. The optical properties of AgNPs, synthesized with DES-based grape pomace extract were analyzed by the UV-visible spectrometer. The absorption spectra of dispersed silver nanoparticles were obtained. Local surface plasmon resonance (LSPR) bands are observed in the visible region of 300–700 nm. The UV-visible spectrum of AgNPs exhibits the maximum absorption at 436 nm after 24, 30, 72, and 120 h after the synthesis of nanoparticles (Figure 6). The data obtained confirm that the reducing ability of the extract based on proline-lactic acid is higher than that of betaine-lactic acid, as evidenced by the higher values of the absorption spectra (1.8 and 1.5, respectively). However, the stability of the systems obtained using betaine-lacticacid-based extracts is higher. The data obtained are consistent with the estimate of electrochemical stability windows of DES depending on water content. The UV-visible spectrum of AgNPs exhibits the maximum absorption also at 436 nm, and the position of the peak remains unchanged despite of the long period of the storage. Analysis of the obtained data indicates that the synthesized systems are stable even after a long period of time, namely, 1 month. It is known from numerous publications that the optical spectra of metal nanoparticles strongly depend on the nature of the metal itself, its geometric shape, size, and the dielectric fluctuation of the medium around the nanoparticles. At the same time, the shape of nanoparticles can be determined by the frequency of the LSPR band within the visible and near-infrared regions of the spectrum. UV spectra of colloidal systems obtained using an aqueous extract of grapes indicate that the peak confirming the formation of nanoparticles is also in the range 400–450 nm.

The color change of the solution during the reaction was observed. However, it can be noted that the intensity of the peak is higher, and it also does not have a clear maximum. The obtained data indirectly indicate that the particle size in such systems is higher and the composition is more polydisperse. The UV-visible spectrum of AgNPs exhibits the maximum absorption also at 436 nm, and the position of the peak remains unchanged despite of the long period of the storage. Analysis of the data obtained indicates that both studied synthesized systems are stable even after a long period of time, namely 1 month. It is known from numerous publications that the optical spectra of metal nanoparticles strongly depend on the nature of the metal itself, its geometric shape and size, and the dielectric fluctuation of the medium around the nanoparticles.

**Figure 7:**
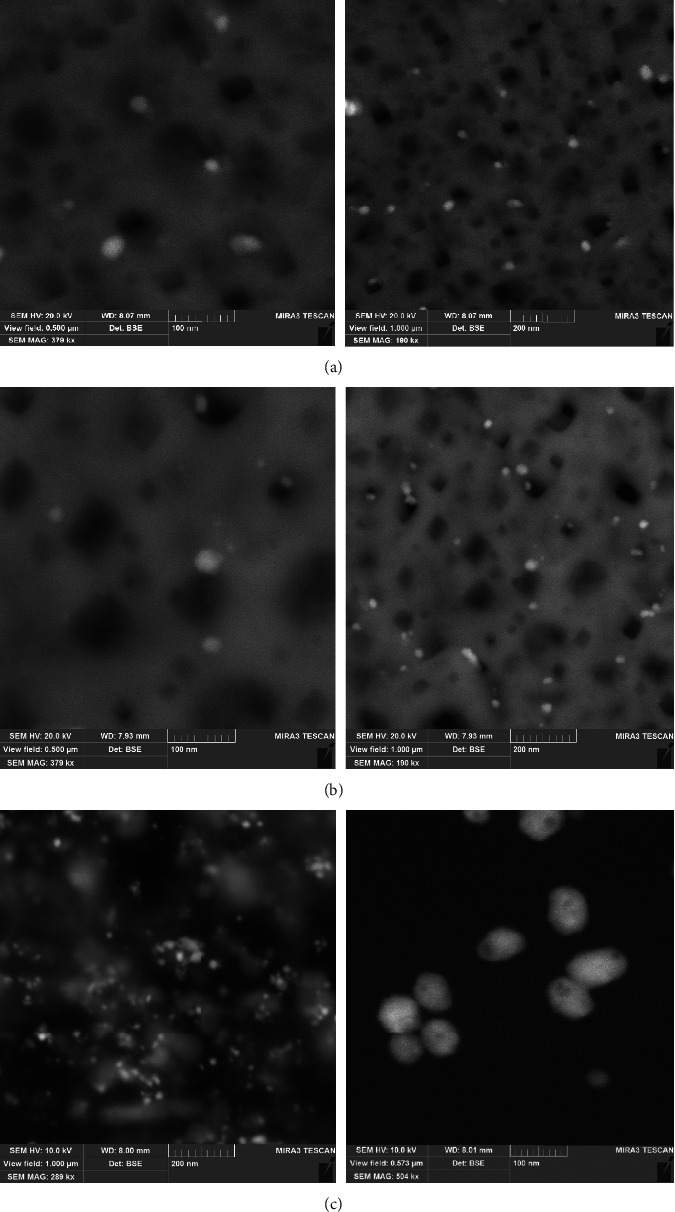
SEM analysis of “green” synthesized AgNPs (a) with grape pomace extract obtained by P-GPE (b), B-GPE (c), and W-GPE (d).

At the same time, the shape of nanoparticles can be determined by the frequency of the LSPR band within the visible and near-infrared regions of the spectrum. Based on the data from work [1], where the analytically calculated extinction spectra of silver nanoparticles are provided, we can suppose that synthesized silver nanoparticles have the shape of flattened spheroids. SEM analysis of synthesized AgNPs confirmed the spherical shape of nanoparticles and allowed to measure an average size that was found to be around 10–15 nm (Figure 7). The SEM images of AgNPs synthesized by W-GPE showed the existence of small spherical nanoparticles with a size range from 10 to 33 nm. However, it should be noted that the nanoparticles obtained by an extract based on DESs, after sample preparation, namely, deposition on a silicon substrate and exposure to air, formed a thin “polymer-like” film. Therefore, it is likely that the particles also look like this, as if in a carrier matrix. The present results indicate that DESs in the extract exerted constructive effects on AgNP synthesis, although the DESs themselves cannot reduce silver ions. Although all systems were either spherical or nearly spherical, the DES-based AgNPs displayed different sizes than the water-based AgNPs. Therefore, it is supposed that DES may play a role by functioning on the surface of the AgNPs to enhance their dispersion. Compared with the water-based GPE, the DES-based GPEs contained lower reducing powers but additionally acts as of capping agents, which is the more important property for the creation of necessary particle nanosize and dispersion with colloidal stability. Since the system based on the extract with B-GPE is more stable, further studies were carried out to evaluate its antimicrobial properties.

**Figure 8:**
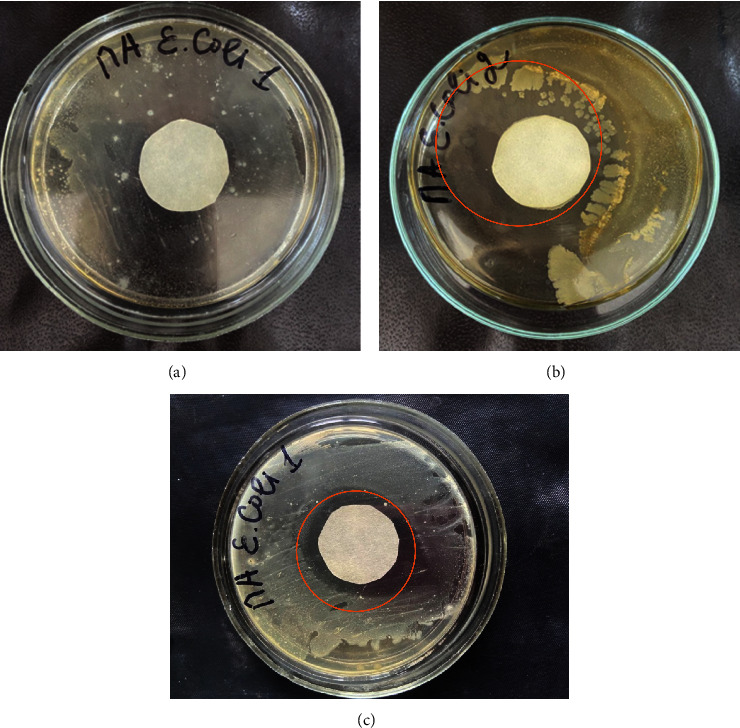
Antibacterial activity of “green” synthesized AgNPs via agar disk diffusion in Gram-negative bacteria (*Escherichia coli*): (a) distilled water, (b) synthesized AgNPs with grape pomace extract obtained by P-GPE, and (c) antibiotic (aztreonam) (Petri dishes were incubated at 37°C for 24 hours).

**Figure 9:**
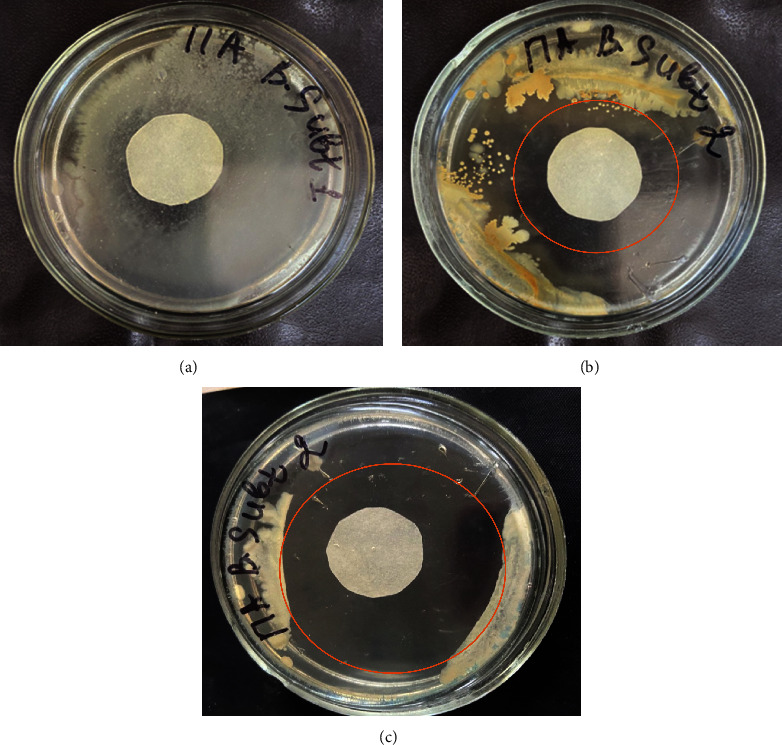
Antibacterial activity of “green” synthesized AgNPs via agar disk diffusion by Gram-positive bacteria *Bacillus subtilis*: (a) distilled water, (b) synthesized AgNPs with grape pomace extract obtained by P-GPE, and (c) antibiotic (aztreonam).

The antimicrobial properties of AgNPs were tested and showed strong antibacterial activity against Gram-negative*Escherichia coli* and Gram-positive bacteria *Bacillus subtilis* (Figures 8 and 9).

**Figure 10:**
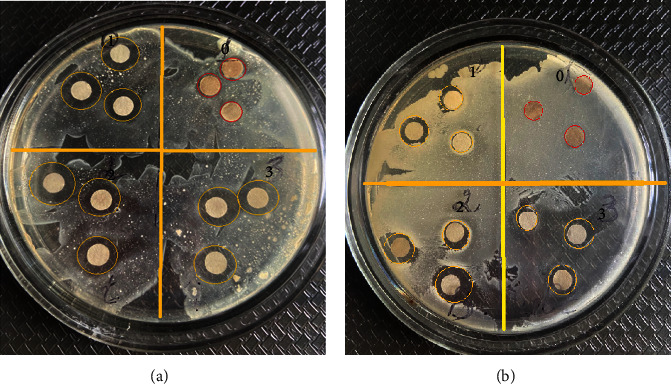
Antibacterial activity of “green” synthesized AgNPs via agar disk diffusion Gram-negative bacteria (*Escherichia coli*) (a) and Gram-positive bacteria *Bacillus subtilis* (b): (1) distilled water, (2-3) synthesized AgNPs with grape pomace extract obtained by P-GPE, and (3) antibiotic (aztreonam) (Petri dishes were incubated at 37°C for 48 hours).

Antibacterial activity was also measured after 48 hours of incubation (Figure 10). The results showed a good inhibitory effect with increasing time. AgNPs shows antibacterial properties due to high effective surface area of the nanoparticles, size, and reactivity. The diameters of the inhibition zones against the target organisms, *Bacillus subtilis* and *Escherichia coli* were 16.03 ± 0.017, 9.10 ± 0.027 mm, respectively. The variation in the antimicrobial effects of AgNPs against Gram-negative bacteria is due to the composition of the cell wall. As expected, the control (water) exhibited no antibacterial efficacy against both the bacteria. Both strains showed notably different susceptibility to the antibiotic tested (Control 2). Our results showed that *B*. *subtilis* bacterial strains were resistant to aztreonam, while *E*. *coli* bacterial strains to a lesser extent. The data obtained are consistent with the detailed results [43].

AgNPs showed more zone of inhibition because the release of Ag^+^ ions from NPs to bind with bacterial enzymes leads to cell death. Due to this, nanoparticles can penetrate through bacterial cells, causing a more extensive zone of inhibition of silver nanoparticles.

**Figure 11:**
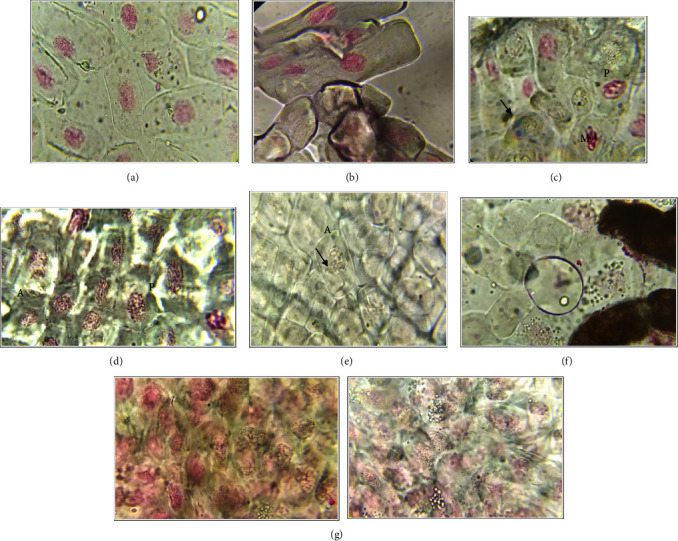
Common view of cells under an optical microscope with a magnification ×1000. (a) Control: normal prophase cells with dyed nuclei contained of visible and tangled chromosomes. (b) AgNP B-GPE 1%: osmotically damaged prophase cells with visible nuclei. (c) AgNP B-GPE 5%: irregular prophase (*P*) and irregular metaphase (*M*) cells with micronucleus (arrows). (d) AgNP B-GPE 10%: prophase (*P*) and irregular anaphase (*A*) cells with apoptosis features under osmotic shock. (e) AgNP B-GPE 1%: irregular anaphase (*A*) cell with not dyed nuclei and a micronucleus (arrow). (f) AgNP B-GPE 5%: irregular prophase cells. Large dark spots on the right side is the deposited of AgNP. (g) AgNP B-GPE 10%: prophase damaged cells under osmotic shock. Prophase cells with apoptosis features under osmotic shock.

The effect of silver nanoparticles and extract obtained by B-GPE on cell division and chromosomal behavior of *Allium cepa* was investigated. To study the proliferative capacity of cells in the primary meristem of *Allium cepa* roots grown in solutions of the studied substances, a study of the length of the roots was carried out. The number of viable cells was estimated under an optical microscope, and the mitotic index was calculated. The viable cells number, their ability to divide, the value of the mitotic index, and their statistical significance are shown in Table 6. It was found that the breadth of the roots of *Allium cepa* are affected the most by the nanoparticles. The results of the visual analysis of cells under a microscope are shown in Figure 11.

In tested solutions, root growth retardation, cell apoptosis and lysis, the formation of micronuclei, and sometimes the deposition of silver salts were observed. Nuclei were poorly stained in damaged cells because the pH of the cytoplasm of only alive cells able to divide is shifted to the acidic side, which promotes active chemical interaction with the dye. In general, apoptosis is the process of programmed cell death used to rid the body of cells that have been damaged beyond repair [40, 44–47].

## 4. Conclusions

This work used a DES-based liquid phase extraction method for extracting “green” compounds from grape pomace:

- The extract was mainly composed of phenolic compounds, with hydroxycinnamic acids being the predominant phenolic compound (45%). To different degrees, the resultant DES-based grape pomace extract showed beneficial effects on the synthesis efficiency of AgNPs. The DESs in the extract exerted constructive effects on AgNP synthesis, although the DESs themselves cannot reduce silver ions. DES-based grape pomace extract can serve as an efficient bio-reducing and stabilizing agent's source for the synthesis of silver nanoparticles. The synthesized AgNPs have a size of∼10–20 nm with a spherical shape, and are of crystalline nature with smooth surface morphology. The spectroscopic studies defined the nanoparticles as face-centered cubic unit structures.
- A more effective stabilizer is the use of a solvent that retains the properties of ionic liquids when mixed with an aqueous solution. Compared with the water-based GPE, the DES-based GPEs contained lower reducing powers, but additionally acts as of capping agents, which is the more important property for the creation of necessary particle nanosize and dispersion with colloidal stability.
- The AgNPs showed higher antibacterial activity against both bacteria (*B*. *Subtilis*), and *E*. *coli.*Root-tips were used for cytogenetic studies using onions (*Allium cepa* L.), which are excellent materials for cytological and genotoxicity studies. Genotoxicity results showed that, by using AgNPs ≤10%, the chromosomes with disturbed abnormalities were not observed and detected, which reflects the not genotoxicity effect of these nanoparticles at this dose. The studied systems significantly inhibited the growth of onion roots, caused toxic effects, such as destruction of the cytoplasm, cell apoptosis, formation of micronuclei, and irregular separation of chromosomes. However, the fact that in the surviving cells division was observed, which according to the MI indicator was not statistically different from the control. Thus, cell viability is preserved even at high concentrations, although it causes visible cytotoxic effects. We planned the next testing of solvent systems and AgNPs in much lower concentrations, which could be expected to cell division stimulation that is important in the medical application of the obtained systems.

## Figures and Tables

**Table 1:** The composition of the studied DESs and their abbreviations for AgNPs synthesis.

DES-nominalcompositions	HBA	Structure of HBA	HBD	Structure of HBD	Molar ratio
DES-1	Lactic acid	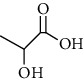	Proline	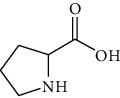	2 : 1
DES-2	Lactic acid		Betaine	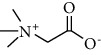	2 : 1
Conventional solvent	Н_2_O				

**Table 2:** Analytical characteristics of the calibration graphs.

Compounds	Linearity range (*μ*g/mL)	Slope (*a*)	Intercept (*b*)	*R* ^2^	Method LOD (*μ*g/mL)	Method LOQ (*μ*g/mL)	RSD (%)
Caffeic acid	0.5–1500.0	40.756 (1.5%)	2648 (25.8%)	0.9997	3.15	10.50	0.52
Gallic acid	0.6–1300.0	96.54 (1.5%)	20.76 (25.6%)	0.9988	1.46	4.88	0.69
(+)-Catechin	0.2–1400.0	186.15 (5.0%)	48.24 (43.5%)	0.9974	3.96	13.20	0.56

**Table 3:** Characterization of the main compounds of the grape pomace extract using their spectral characteristics in HPLC-DAD (Rt, *λ*max, and MS2).

Peak	Rt/min	Mode of ionization [M+H]^+^/[M−H]^−^	Fragments MS2	UV-Vis max	Compound	Percentage		
						Solvent		
						DES-1	DES-2	Water
*Anthocyanins*								
9	15.2	465/—	303	277, 560	Delphinidin 3-O-glucoside	5.1	4.7	2.4
10	16.4	595/—	449, 287	280, 540	Cyanidin-3-O-glucoside	2.7	2.1	1.5
11	16.6	—/493	311	278, 528	Malvidin 3-O-glucoside	7.1	6.4	5.7
12	17.1	479/—	317	278, 527	Petunidin 3-O-glucoside	6.5	6.0	4.7

*Phenolic acids*								
1	1.8	—/169	125, 107, 97, 79	325	Gallic acid^*∗*^	10.8	10.4	11.2
2	4.7	—/153	109	325	Protocatechuic acid	5.2	4.1	4.9
3	5.0	—/137	93	325	p-Hydroxybenzoic acid	3.2	2.8	3.0
4	5.2	—/295	163	325	Coutaric acid	2.7	1.8	2.1
5	6.4	—/179	135	325	Caffeic acid^*∗*^	8.7	6.5	12.8
6	7.3	—/289	245	325	Epicatechin	10.2	8.1	5.2
7	7.7	—/197	153/182	325	Syringic acid	4.2	4.0	5.8
8	8.0	—/163	119	325	p-Coumaric acid^*∗*^	3.1	2.7	3.0

*Anthoxanthins and stilbenes*								
13	20.3	—/289	245	276	(+)-Catechin^*∗*^	9.5	8.1	5.6
14	20.5	—/463	300, 255, 151	354	Quercetin-3-O-glucoside	1.8	1.5	1.4
15	23.8	—/609	300	354	Quercetin-3-O-rutinoside	4.8	4.0	3.2
16	24.5	—/447	284, 255, 227	354	Kaempferol-3-O-glucoside	3.5	3.0	2.7
17	25.0	—/301	301, 151	254	Quercetin^*∗*^	4.3	3.7	2.5

^*∗*^Verified with pure standard. Rt: retention time.

**Table 4:** Concentration of predominant compounds (*μ*g/g).

Extracts	Caffeic acid	Gallic acid	(+)-Catechin
DESs-1	164.5	792.4	511.3
DESs-2	138.1	721.4	454.1
Water	193.2	825.2	279.7

**Table 5:** Total phenolic and flavonoid contents in grape pomace extracts.

Extracts	The total phenolic (TPC)	The total flavonoid (TFC) content
DES-1	92.15 ± 1.75	51.36 ± 1.22
DES-2	80.75 ± 1.75	47.41 ± 1.22
Water	45.10 ± 1.75	12.15 ± 1.22

**Table 6:** Number of alive active cells, their MI calculations and difference validity between samples and control.

Samples	Number of alive active cells (%)	MI, % (mean ± mse^*∗*^)	Td, difference validity by Student's *t*-test	Standard value of difference validity Student's *t*-test for *β* = 0.95	Conclusion about difference validity in compare with control
Сontrol	98	32.12 ± 0.53	0.538	2.1	Unvalid
DES 1%	67	23.2 ± 0.76	0.986	2.1	Unvalid
DES 5%	68	30.21 ± 0.66	0.236	2.1	Unvalid
DES 10%	54	30.09 ± 1.5	0.121	2.1	Unvalid
AgNPs 1%	72	34.14 ± 0.75	0.241	2.1	Unvalid
AgNPs 5%	66	37.53 ± 0.78	0.597	2.1	Unvalid
AgNPs 10%	65	42.02 ± 0.671	1.215	2.1	Unvalid

^∗^mse: mean squared error or mean squared deviation.

## Data Availability

The HPLC profile, SEM and antibacterial activity data, reducing powers, and AOA data used to support the findings of this study are available from the corresponding author upon request (vorobyovavika1988@gmail.com-Victoria Vorobyova).
